# Bioinformatics Approaches in the Development of Antifungal Therapeutics and Vaccines

**DOI:** 10.2174/0113892029281602240422052210

**Published:** 2024-05-16

**Authors:** Vaishali Ahlawat, Kiran Sura, Bharat Singh, Mehak Dangi, Anil Kumar Chhillar

**Affiliations:** 1 Centre for Biotechnology, M.D. University, Rohtak, Haryana, India;; 2 Centre for Bioinformatics, M.D. University, Rohtak, Haryana, India;; 3 Department of Biotechnology and Central Research Cell, MMEC, Maharishi Markandeshwar (Deemed to be University), Mullana-Ambala, Haryana-133207, India

**Keywords:** Antifungal resistance, drug repurposing, reverse vaccinology, pharmacomicrobiomics, multidrug resistance, pandrug resistance

## Abstract

Fungal infections are considered a great threat to human life and are associated with high mortality and morbidity, especially in immunocompromised individuals. Fungal pathogens employ various defense mechanisms to evade the host immune system, which causes severe infections. The available repertoire of drugs for the treatment of fungal infections includes azoles, allylamines, polyenes, echinocandins, and antimetabolites. However, the development of multidrug and pandrug resistance to available antimycotic drugs increases the need to develop better treatment approaches. In this new era of -omics, bioinformatics has expanded options for treating fungal infections. This review emphasizes how bioinformatics complements the emerging strategies, including advancements in drug delivery systems, combination therapies, drug repurposing, epitope-based vaccine design, RNA-based therapeutics, and the role of gut-microbiome interactions to combat anti-fungal resistance. In particular, we focused on computational methods that can be useful to obtain potent hits, and that too in a short period.

## 1 INTRODUCTION

About 3-5 million fungal species have been estimated to exist on our planet, of which about 300 species are pathogenic to humans [1]. *Aspergillus*, *Cryptococcus*, *Pneumocystis*, and *Candida* are the four major genera that cause lethal infections [2]. Additionally, *Candida* and *Aspergillus* have been identified to interact synergistically with the COVID-19 virus [3]. Treatment for mycotic infections relies on five major classes of drugs: echinocandins, polyenes, azoles, allylamines, and antimetabolites [4]. Although these drugs are effective in many cases, their therapeutic efficacy is limited because of their high toxicity and frequent development of resistance to therapeutics [5]. Despite the advancements in the mega science of mycology, the development of novel antifungal therapeutics remains a challenging, time- consuming, expensive, and inefficient process. This review emphasizes how the integration of bioinformatics and multi-omics approaches complement the development of new therapeutic products.

The fungal pathogens develop strategies to escape the host immune system and confer resistance to protective antifungal response. One such strategy is shielding the pathogen-associated molecular patterns (PAMPs) with different molecules, such as β-1,3-glucan, by the outer mannan layer, preventing its interaction with dectin-1. Dectin-1 is a pattern recognition receptor (PRR) on host immune cells and mediates antifungal cellular responses [6]. In dimorphic fungi, β-1,3-glucans (more immunogenic) are converted into α -1,3 glucans (less immunogenic) during a change in morphology from filamentous form to yeast form. Eng1 protein secreted by *Histoplasma caspulatum* has glucanase activity, which reduces as β-1,3-glucan on yeast cell wall [7].

Numerous mechanisms related to the development of antifungal resistance have been identified, including alteration or overexpression of antifungal targets, reduction in the intracellular drug concentration due to upregulation of multidrug transporters, biofilm formation, and activation of stress responses [8]. These processes are influenced by multiple factors like drug misuse, lack of strong regulatory measures, improper sewage disposal, and low-quality medicine and non-specific medications, causing the emergence of drug-resistant microbes [9]. The long-term use of antifungals can result in serious adverse drug effects (ADEs). The triazoles (itraconazole, posaconazole, voriconazole, fluconazole, and isavuconazole) has recently gained attention for its ADEs. The use of posaconazole causes an elevation in liver enzymes leading to hepatotoxicity, while voriconazole and isavuconazole have the highest risk of nervous disorders. The echinocandins consist of three approved drugs: micafungin, caspofungin, and anidulafungin. Furthermore, all three drugs are similar in their chemical structure, but it was found that micafungin and caspofungin have the highest incidences of subcutaneous tissue and skin disorders [10]. Amphotericin B, belonging to class polyenes, was the first antifungal drug approved by the FDA for the treatment of mycosis. The major drawback of Amphotericin B reported was its toxicity, notably nephrotoxicity, which causes kidney damage [11]. The other ADEs related to amphotericin B include subcutaneous tissue and skin disorders, a decrease in potassium levels, and respiratory and gastrointestinal disorders [10].

Antifungal vaccines could be an alternative treatment to eradicate fungal infections. Conventional methods of vaccine development rely on the growth of the pathogen in laboratories and the purification of antigenic proteins that can serve as potential vaccine candidates. These traditional approaches, besides being time-consuming and of low yield, have failed in several instances, such as in the cases of non-culturable/cultivatable pathogens [12]. Advances in bioinformatics facilitate the birth of a rationalized strategy known as reverse vaccinology (RV) [13]. The RV approach exploits the whole genome of the pathogen and searches for putative immunogenic targets. The basic idea behind this approach was B and T cell receptors recognize the predicted antigenic determinants and evoke both humoral and cell-mediated immune responses [14].

The restricted scope of currently available antifungals calls for finding new therapeutic approaches. Integrated genomics, transcriptomics, proteomics, and bioinformatics aid in the advancement of therapeutics for various infectious diseases. Various computational strategies have been employed to investigate how drug candidates interact with target proteins and elicit a therapeutic response impacting biological pathways and functions. The development of better diagnostic tools and strategies that allow targeted use of antifungals is essential to promote drug effectiveness. This article provides insight into the use of bioinformatics and computational approaches for novel therapeutic discoveries.

## 2 BIOINFORMATICS IN THE IDENTIFICATION OF POTENTIAL THERAPEUTIC TARGETS

### 2.1 Mechanistic Targets for Available Antifungals and Resistance Mechanism

Based on the action mechanisms, the existing classes of drugs have three main targets: (i) inhibition of ergosterol biosynthesis, (ii) disruption of the fungal membrane, and (iii) inhibition of macromolecule synthesis [15]. Azoles block the activity of 14-sterol demethylase, an enzyme belonging to the Cytochrome P-450 family, that plays a role in ergosterol production. This causes the depletion of ergosterol and the accumulation of toxic sterol intermediates that lead to the loss of membrane integrity, as ergosterol is the main component of the fungal cell membrane, which results in cell death [16]. The prevalence of resistance to azoles happens due to mutations in the target gene ERG11 and overexpression of efflux transporters that cause azole molecules to escape outside the cell [8]. Polyenes, another class of antifungal drugs, interact with the fungal membrane and target a vital molecule-ergosterol [17]. The binding of polyenes to ergosterol facilitates the leakage of intracellular ions that disrupt the membrane potential and active transport mechanism. Amphotericin B belongs to the class polyenes, and it was the first antifungal discovered. The major drawback of Amphotericin B reported was its toxicity, notably nephrotoxicity, which causes kidney damage [11]. Flucytosine (5-fluorocytosine) is an antimetabolite that targets DNA and RNA synthesis in fungi. Once it enters the fungal cell, it is metabolized into 5-fluorouracil, a pyrimidine analog that can be disincorporated into DNA and RNA. Two common side effects attributed to flucytosine are hepatotoxicity and hematological toxicity [18]. Another major antifungal category is Echinocandins, which block the activity of 1,3- β-d-glucan (BDG) synthase. BDG synthase is an enzyme responsible for the synthesis of 1,3- β-d-glucan, which is one of the main structural elements of the cell wall in most fungi but is absent in mammalian cells, therefore making it a perfect target for antifungals [19]. However, mutations in the gene FKS1, which encodes a catalytic subunit of glucan synthase, serve as the site of drug resistance [20].

### 2.2 Computer-aided Target Discovery

The currently available antifungals either target cell wall synthesis, ergosterol synthesis, or ergosterol itself. However, these targets constitute a minor fraction of potential therapeutic targets encoded by the fungal genome [21]. To improve therapeutic success, pharmacological molecules interact with specific targets, therefore, identification of new targets and target validation are of utmost importance for the development of new therapeutic molecules. There are two basic criteria for a gene to function as a therapeutic target. First, pathogen survival and growth must rely on that gene. Second, the homolog of the candidate gene must not be present in mammals. Traditionally, target identification relies on wet lab experiments and is cost-ineffective, time-consuming, and has low accuracy. In the multi-omics era, computer-aided target identification is overcoming the limitations of conventional methods. Bioinformatics has created a paradigm shift in the identification of novel drug targets and facilitates the process of drug discovery. Novel targets can be uncovered using two methods: A) Comparative genomics, which includes a comparison of host and pathogen metabolic pathways. B) Network-based approach, is useful for constructing the endogenous signaling, regulatory, and metabolic pathway with which the novel drug target can interact [22, 23].

#### 2.2.1 Comparative Genomics Approach

The comparative genomics approach is based on the fact that candidate drug targets are key components in metabolic pathways and are crucial for pathogen survival. Moreover, the comparative genomics approach, combined with metabolic pathways analysis, yields the proteins specified to the pathogen and is followed by the subtractive proteomics approach, which narrows the selection for target identification.

Firstly, all the existing metabolic pathways from both the host and pathogen are collected and compared using databases like KEGG or MetaCyc. In addition, common metabolic pathways are removed, and genes as well as enzymes belonging to unique metabolic pathways are identified. In the second step, protein sequences for the enzymes involved in pathogen-specific pathways are retrieved in FASTA format from the UniProt database and subjected to homology searches using the BLAPSTp tool. Non-homologous pathogen enzymes are identified using BLAST results that have no hits with host enzymes [23]. Finally, resultant proteins are prioritized using several parameters, including 1) the selection of essential proteins, virulent proteins, and resistance proteins, 2) Subcellular localization (cytoplasmic proteins are more suitable as drug targets), 3) the removal of proteins already existing as drug targets for novelty, and 4) Drug ability and toxicity analysis. The novel/potential drug targets identified using the above-mentioned techniques need to be further subjected to structure generation and validation, which the present article does not factor into its scope. Fig. (**1**) shows a workflow for *in-silico* identification of novel targets using a Comparative Genomics approach.

#### 2.2.2 Network-based Approach

The concept of network graph theory explores the biological network by mapping all the relevant data through data mining. This helps to identify the functional concept in the network and identify the potential targets [24]. The integration of such huge biological datasets requires system biology tools and computational algorithms together with the use of functional genomic and network analysis databases. Cytoscape [25] and Gephi [26] are the two popularly used tools for complex network analysis. GeneMANIA is a web-based tool for analyzing gene lists [27]. These tools identify the sub-networks and regions of similarity and dissimilarity to interpret the interactions within the network. Paolini *et al.* first developed the drug-target network based on 200,000 compounds with more than 500,000 bioactivities by linking proteins through chemical spaces [24].

The functional component in the biological network is depicted as a node, and any connection between the nodes, which can be physical or functional interactions is termed as edge. Different types of networks include gene interaction networks, protein-protein interaction networks, mi-RNA-mRNA interaction networks, signal transduction networks, metabolic networks, and genetics interaction networks. Table **1** tabulates the parameters for analyzing the general structure of biological networks [28, 29]. In general, network-based approaches require an in-depth knowledge of the interaction network and, therefore, require pathway enrichment analysis to identify the potential drug target. Fig. (**2**) describes the workflow of the network-based approach.

Recently, Robin *et al.* proposed three promising therapeutic targets against *Cryptococcus gattii* using comparative genomics and subtractive approach that are- Mitochondrial distribution and morphology protein 10 (MDM10), osmolarity two-component system, phosphorelay intermediate protein YPD1 (YPD1), and mitochondrial distribution and morphology protein 34 (MDM34, MMM2) [30]. However, the study is completely based on computational analysis and still needs to be confirmed experimentally. Rrp9 (U3 small nucleolar ribonucleoprotein associated protein) is a promising drug target against *Candida albicans* based on *in-silico* studies. Docking studies revealed that it shows binding affinity with dicyclomine, which targets signal transduction genes and inhibits virulence factors in *C. albicans* [31]. Computational studies revealed that 5 protein-coding genes, namely His6, PabaA, FasA, FtmA, and erg6, can act as putative drug targets against *Aspergillus fumigatus* [32]. However, elucidation of the 3D structure of these targets is lacking till now.

## 3 RECENT THERAPEUTIC APPROACHES FOR COMBATING ANTIFUNGAL RESISTANCE

### 3.1 Advancements in the Drug Delivery System

Despite the available antifungal therapeutics, the prevalence of fungal infections is still increasing due to the development of multidrug resistance to existing antifungal drugs, and the foremost reason for the development of drug resistance is found to be associated with suboptimal drug concentration and non-specific cell targeting [33, 34]. Furthermore, novel antifungal therapies show less efficacy due to the insufficiency of the suggested route of administration, lack of controlled clinical trials, or high cost of production compared to conventional antifungals [35]. The development of the drug delivery system based on nano-formulations provides insight to overcome these limitations. However, most of the available drugs are hydrophobic, which reduces their solubility and bioavailability, causing pharmacokinetic problems. However, the pharmacokinetic profile can be improvised by the covalent conjugation of drugs with the polymers [36]. Efforts have been made to optimize the compatibility between drugs and nanoparticles using the *in-silico* approach, which is time-effective and augments drug loading, retention, and stability. Molecular simulations are the ideal technique to improve the design of drug delivery devices and are driven by long-range non-covalent interactions. Simulations serve as a “computational microscope,” which provides information that is difficult to get experimentally, such as the influence of molecular interactions on crucial parameters like release rate, drug delivery device's responsiveness to external stimuli, and interactions between nanoparticles and biological material [37]. Several Databases/Tools are available to design nanoparticle-based drug delivery systems (Table **2**). However, so far, there is no currently stored information regarding the 3D structure of nanomaterials and their correlation with physicochemical properties and toxicity, which brings about the need to build a database regarding such information.

### 3.2 Combination Therapy

Combination therapy using multiple drugs, is a promising therapeutic strategy, improving the combined molecules' efficacy, reducing toxicity, and combating antimicrobial resistance [38]. Clinical studies have demonstrated the effectiveness of combination therapy in various instances. Shaban *et al.* demonstrated that Carvacrol, a monoterpene phenol, shows both additive and synergistic effects when combined with antifungal drugs: nystatin fluconazole, caspofungin, and amphotericin B against *C. auris* [39]. Terbinafine (TEF) and azoles show synergistic effects; azoles target the plasma membrane, increasing TEF absorption [40]. Various combinations of plant natural products and existing antifungal drugs have been investigated for combating antifungal resistance, such as Brazilian Red Propolis and *A. Sellowiana* in combination with fluconazole act synergistically against *C. parapsilosis* and *C. glabrata*. Propolis acts on the cell wall and facilitates the penetration of fluconazole inside the cells [41]. Recently, it has been reported that ribavirin works synergetically with caspofungin against *C. albicans* and can be effective in treating *C. albicans* infections [42].

There will be millions of combinations for the thousands of FDA-approved drugs, and the systematic high-throughput screening of all possible drug combinations is time-consuming and challenging, therefore, there is delimited knowledge of effective drug combinations [43]. There are many unanswered questions, like which two molecules in combination would be optimal or at what concentration they will work as a better therapeutic agent? What endpoint is relevant? What percentage of populations are likely to get leverage? How can combination therapy be justified over monotherapy? How to counterbalance the production cost and the chances of potentially increased toxicity of this approach?

Computational strategies enable *in silico* screening of combination effects. The network-based approach is one such strategy that offers novel insight to explore the “multiple-drug, multiple targets” paradigm aiming at modifying multiple disease proteins within the same disease module while minimizing toxicity profiles [44].

The pharmacokinetic and pharmacodynamic properties of drugs and the appropriate drug dosage in combination can be quantified using mathematical modeling [45, 46]. The binding effect of each drug involved in combination therapy is determined using the MD simulations, which also suggest the possible outcome of the allosteric binding of other drugs, whether the drugs in combination show synergism or antagonism [47].

Various databases have been generated on combination therapy, such as the Drug combination database (DCDB) [48], drug-drug interaction (DDI) [49], Antifungal synergistic drug combination database (ASDCD) [50], DrugComb (DB) [51]. Some freely available software and tools that have been developed for analyzing combination data based on machine learning techniques are Combenefit [52], SynergyFinder [53], Synergy [54], and SynToxProfiler [55]. However, the lack of available input data is still considered a major limitation for the computational design of combination therapies that demands attention for bringing in better results of combination therapies.

### 3.3 Drug Discovery

#### 3.3.1 De Novo Drug Development

Developing novel drugs with required pharmacological activity is crucial for maintaining the development pipeline. Computer-Aided Drug Design is an efficient tool to expedite the drug discovery process and relies on information regarding the receptor (target) and its binding ligand. The two different approaches in CADD are Structure-Based Computer-Aided Drug Design (SB-CADD) and Ligand-Based Computer-Aided Drug Design (LB-CADD) [56]. SB-CADD approach includes the following steps: (1) Mining data: Various databases have been developed to extract information about protein structural data, drug interactions, side effects, metabolic pathways, protein-protein interactions/networks, drug targets, *etc*. (2) Protein structure prediction: 3D structures of proteins can be predicted computationally using methods such as homology modeling and *de novo* modeling. However, the former is the best-suited method and the most accurate [57]. Other methods for the detection of 3D structures of protein include X-ray crystallography, NMR, and Electron microscopy [58]. (3) Molecular docking and Molecular dynamic (MD) simulations: Molecular docking allows the prediction of interaction between a drug candidate and target protein (receptor) to make a stable complex [59]. Various docking programs have been developed till now, and among all software, Autodock Vina, MOE-Dock, and GOLD give the best scores with their algorithms [60]. Molecular docking is insufficient for understanding the behavior of a drug in the actual physical system, as proteins are dynamic and exist in different conformational states. MD simulations are an advantageous technique to overcome the shortcomings of molecular docking [61]. LB-CADD: A ligand-based approach is implemented when the 3D structure of the protein or target molecule is unavailable [62]. This approach elucidates the relationship between the structural and physicochemical properties of the compound and its biological activity [63]. The two most widely used computational strategies in the LB-CADD approach are Quantitative structure-activity relationships (QSAR) and pharmacophore modeling [64]. While the 3D QSAR pharmacophore approach incorporates the chemical properties of both the most active and inactive compounds together with their biological activity, pharmacophore modeling solely makes use of the common chemical features found in the most active compounds. DrugRep is a web server for re-profiling drugs that achieves its task using both receptor-based screening and ligand-based screening. The cavity detection approach detects the possible binding pockets of receptors and performs batch docking using AutoDock Vina [65]. However, discussing the details of these approaches is not in the scope of this article.

#### 3.3.2 Drug Repurposing

The re-profiling of existing drugs, as compared to the traditional drug discovery approach or new drug designing is cost-effective and time-saving with additional benefitssuch as lower chances of failure in the later stages of clinical trials [66]. Multi-omics era and bioinformatics analysis provide insight into drug repurposing [62]. Antifungal effects of various non-antifungals (antitumor and antimicrobial agents) can be uncovered using the drug repurposing strategy. Various anti-bacterial drugs such as aminoglycosides, macrolides, tetracyclines, quinolone peptides, and others, including rifampicin and linezolid, are also known to possess antifungal activities [67]. It has been recently reported that atorvastatin, an inhibitor of HMG-CoA reductase, a lipid-lowering drug, is confirmed to have antifungal activity in fluconazole-resistant *Candida albicans* [68].

Computational drug repurposing have been classified into drug-centric (similar drugs have similar pharmacological effects) and disease-centric (similar disease needs the same therapies). Current *in-silico* approaches that have been developed in the context of drug repurposing are of three types: (1) target-driven repurposing, (2) genome-wide repurposing, and (3) Literature-driven repurposing [69].

##### 3.3.2.1 Target-driven Repurposing

The affinity of drug molecules to more than one target is the key notion behind target-driven repurposing [70]. Target-driven repurposing exploits the drug libraries available for high-throughput screening, followed by virtual screening such as docking or ligand-based screening. This approach can screen nearly all drug compounds with known chemical structures [71]. Based on protein targets, new indications are identified by linking a drug to a specific disease [69].

##### 3.3.2.2 Genome-wide Repurposing

The advancement in genome-wide metrics has made it possible to repurpose FDA-approved drugs for treating heterogeneous diseases [72]. The Online Mendelian Inheritance in Man (OMIM) and the Gene Expression Omnibus (GEO) are two publicly available repositories that enable a systemic survey of disease similarity within the framework of the genome. The drug-target interaction networks represent another domain of genome-wide repurposing. It exploits the disease omics data because disease pathways can be constructed using network analysis [69].

##### 3.3.2.3 Literature Driven Repurposing

The literature-driven repurposing, or “text mining,” leverages the huge scientific literature on drugs and disease [71]. Bioinformatics and chemoinformatics tools combined with the text mining approach led to novel discoveries systemically. Several information sources or databases are available for indication discovery such as PubMed and OMIM [69].

### 3.4 Drug-microbiome Interactions

The gut microbiome (GM) and drug interactions share a reciprocal relationship. GM can interfere with drug metabolism and, hence, can increase, decrease, or toxify the drug efficacy to a clinically significant level. On the contrary, drug intake may also alter the composition of gut microbiota, which, in turn, may affect the individual's health and other drug responses [73]. Drug metabolism by GM of over 180 orally administered drugs has already been reported, and non-oral administered drugs are under research [74]. Human GM shows inter-individual variation and acts as unique fingerprints [75]. A new field, “Pharmacomicrobiomics,” has been proposed, to investigate the interplay between GM variation and drug pharmacodynamics and pharmacokinetics [76]. Drug absorption, distribution in the body, metabolism, and elimination (ADME) are the four fundamental processes studied in the field of pharmacokinetics. GM interaction with antifungal drugs has been reported in the literature. Fluconazole administration is the most widely used antifungal, which impacts the gut microbiome composition [77]. Hence, the study of the drug-microbiome interactions can prove to be a milestone in antifungal treatment.

Table **3** depicts some available databases that contain information about drug-microbiome interactions. DrugBug is the only tool available to predict the susceptibility of the drug to get metabolized by the GM. In addition, DrugBug was developed using a machine learning technique based on the structural similarity of drugs, as drugs with certain functional groups are more prone to metabolism by the GM [78]. Currently, the use of the *in-silico* approach in understanding drug-microbiome interactions lags behind other areas. The emergence of next-generation sequencing and advancements in the characterization of GM provide a lot of the latest information to create datasets and develop novel computational pipelines using these datasets. Moreover, the composition of GM varies in every individual due to numerous factors like population difference, age, diet, genome, presence of disease, lifestyle, and gender [79]. Hence, the universalization of these studies is still far from being achieved.

### 3.5 RNA-based Therapeutics

RNA molecules are used as a drug or a vaccine to generate a therapeutic response in experimental organisms which brings RNA-based therapeutics to the forefront as an emerging source of treatment option for different fungal infections. The core concept of RNA therapeutics is the manipulation of protein function and/or production. This can be accomplished by either directly targeting proteins, interfering with the RNAs that encode the necessary proteins, or supplying the genetic instructions for protein synthesis [83]. RNA- mediated gene silencing is a well-conserved phenomenon and has been investigated in a diverse group of fungi. There used to be so many limitations on the practical implications of RNA-based methods, such as rapid therapeutic deterioration, specificity to inhibit fungal pathways only, crossing the cell envelope barrier, and challenges in facilitating RNA escape from the endosome [84]. Some frequently used strategies to overcome the challenges felt in RNA-based therapeutics are nanoparticle-based delivery [85], chemical modification to prevent deterioration and decrease immunogenicity [86], and the use of aptamer as a delivery carrier to increase specificity [87]. Based on machine learning, various bioinformatics tools and servers have been developed which facilitate the designing of RNA-based therapeutics and are discussed in the next section.

In *Aspergillus nidulans*, the siRNA shows an *in-vitro* gene silencing effect targeting ornithine decarboxylase (ODC), a fungal polyamine gene essential for fungal growth and development [88]. RNAi mechanism was successfully tested in *Aspergillus fumigatus* against ALB1/PKSP and FKS1 genes [89].

imRNA is a server for designing immunomodulatory single-stranded RNA to develop RNA-based therapeutics. In addition to it, this server may also identify minimum mutations required to decrease the immunomodulatory potential of a given RNA sequence. Computer-aided designing of siRNA can also be done using this server [90]. AptaBlocks is a computational approach that aids in designing RNA complexes and improvising RNA-based drug delivery systems [91]. PFRED is another computational platform for designing antisense oligonucleotides and siRNA [92]. Si-Fi (siRNA Target Finder) is yet another tool available online for designing siRNA [93]. Various link prediction models have been developed, such as GKLOMLI for inferring miRNA–lncRNA interactions [94], SPRDA predicts piRNA associated with diseases [95], and AMDECDA predicts circRNA-disease association [96].

## 4 MODERN VACCINE DEVELOPMENT

Traditional methods of developing vaccines come at a huge cost in terms of time and money [97]. Artificial intelligence-driven immunology research has led to the emergence of immunoinformatics as the field of study [98]. The use of immunoinformatics tools in designing vaccines nowadays has also facilitated a rationalized strategy known as “Reverse Vaccinology (RV). The basic premise of the RV approach is to search for immune-dominant epitopes that can be recognized by B and T cell receptors, known as B cell epitopes (BCEs) and T cell epitopes (TCEs), respectively, which evoke both humoral and cellular immune response [14]. The most widely used bioinformatics tools available for the prediction of epitopes are tabulated in Table **4**. Promiscuous antigenic proteins are filtered using the subtractive proteomics approach [99], and the epitope selection list is narrowed down based on antigenicity, allergenicity, immunogenicity, druggability, virulence, self-tolerance, immune boosting potential, toxicity, conservancy, *etc* [100]. Since the single-epitope-based vaccine has low immunogenicity and antigenicity, hence the multi-epitope vaccine construct was favored. The epitopes and adjuvant can be fused using appropriate linker selection, and the final vaccine construct can be simulated and evaluated before the experimental validation [14]. RV approach has been successfully applied to build effective subunit vaccine candidates against emerging strains of mycobacterium, peptide vaccines based on essential genes and virulent genes against bacterial infections, subunit vaccines against the Zika virus, and many others against coronaviruses [101].

## CONCLUSION

Despite advancements in antifungal treatment, the prevalence of infections is still increasing, resistance to the existing antifungal drugs remains a major concern, and the goal of achieving control over fungal diseases is constantly pushed further. Of course, there has been tremendous progress in the development of novel antifungal drugs, but it may take many years from discovery to clinical use. For this reason, it is important to optimize existing molecules and develop novel combinations and alternative therapies to prevent and treat mycosis. Computer-aided drug designing, nano-modeling tools, drug repurposing, and immunoinformatics approaches can change the paradigm of therapeutic development. Moreover, the interactions between the microbiome and drug metabolism need to be explored further to improve drug efficiency. To conclude, it can be said that bioinformatics or computational approaches have the immense potential to accelerate the process of identification of more efficient drug/vaccine candidates and facilitate the development of antifungal therapeutics.

## AUTHORS’ CONTRIBUTIONS

The authors confirm contribution to the paper as follows: V.A., K.S., B.S., M.D. and A.K.C. contributed to the research design and implementation, as well as the data analysis and manuscript writing.

## Figures and Tables

**Fig. (1):**
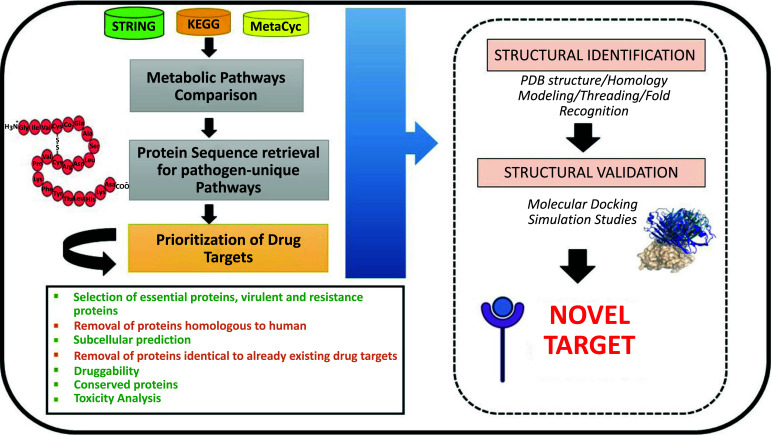
The computational workflow for the identification of novel drug targets using comparative genomics.

**Fig. (2):**
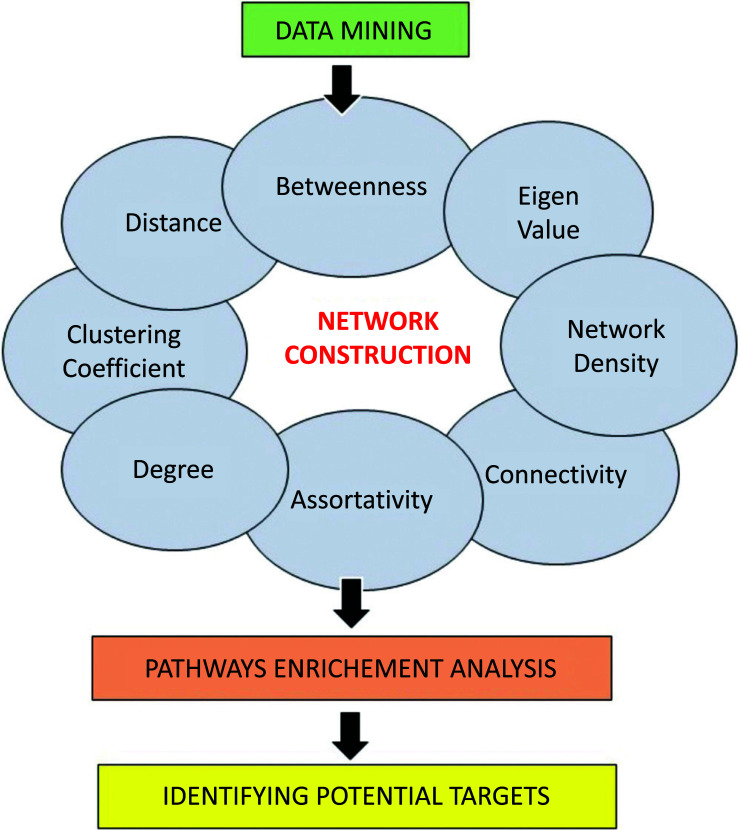
The computational workflow for the identification of novel drug targets using a network-based approach.

**Table 1:** Description of the parameters used in biological network analysis.

**Parameters**	**Description**
Network density	Maximum number of edges connecting each node to each other.
Degree	The property of the node to interact with other neighboring nodes.
Betweenness	The frequency with which the distance between any pair of nodes passes through that node.
Distance	The shortest path length between two nodes.
Clustering coefficient	It measures the interconnectivity of its neighbors.
Connectivity	Minimum number of elements that need to be removed to disconnect the leftover nodes from each other.
Assortativity	Measures the correlation coefficient of degree between pairs of linked nodes.
Eigen value	It is the measure of the influence of nodes in a network.

**Table 2:** Databases/tools available to design nanoparticle-based drug delivery systems.

**Name**	**Type**	**Description**	**URL**
**Nanowerk**	Database	Currently available nanomaterials (about 4500)	http://www.nanowerk.com/
**ENanoMapper**	Database	Provides toxicology data	http://data.enanomapper.net/
**NBI knowledge base**	Repository	Data on nanomaterial characterization, biological interactions, and synthesis methods	http://nbi.oregonstate.edu/
**Nanomaterial Registry (NR)**	Repository	Physiochemical properties of nanomaterials and their biological interactions	http://naomaterialregistry.net/
**PubVINAS**	Tool	Nano-modeling tool	http://www.pubvinas.com/

**Table 3:** Drug-microbiome interaction databases.

**Name**	**Description**	**References**
**Microbe Drug Association Database (MDAD)**	Contains experimentally supported information about drug-microbe interaction	[80]
**PharmacoMicrobiomics database**	Classify drug-microbe interaction based on microbial taxa and body site	[81]
**Microbiota- Active Substance Interaction Database (MASI)**	Provide information about the abundance of GM, drug impact on GM, and vice-versa	[82]

**Table 4:** The most widely used bioinformatics tools available for the prediction of epitopes.

**MHC-I Binding Prediction Tools**		**MHC- II Binding Prediction Tools**		**Linear B-cell Epitope Prediction Tools**		**Conformational B-cell Epitopes Prediction Tools**	
*IEDB* *NetCTL* *MHCpred NetMHC nHLAPred* *CTL-Pred* *SVMHC*	*RANKPE* *BIMAS* *MAPPP* *ProPred SYFPEITIPREDEP* *MHCPEP*	*IEDB* *NetMHC-II MHCpred* *MetaMHC MetaSVMP, Propred-II* *RANKPEP PREDIVACEpiDOCK Consensus*	*SYFPEITH,* *BIMAS CTL-pred* *EpiTOP* *MHCPEP EpiVax,* *PREDEPP TEPITOPE* * EPIPREDITEpiMatrix*	*Bepipred BCpred* *ABCpred* *Pcipep BCEpred* *Igpred*	*BepiTope* *PrediTop PEOPLE LBtope* *SVMTrip* *COBEproEPMLR*	*Discotope* *Ellipro* *CBTope* *Epitope* *BEPro* *CEP* *SEPPA*	*CED* *EPITOME* * MAPOTODEEPCES* * EPSVR EPMETA*

## LIST OF ABBREVIATIONS

ADEs: Adverse Drug Effects
ASDCD: Antifungal Synergistic Drug Combination Database
BCEs: B Cell Epitopes
DCDB: Drug Combination Database
DDI: Drug-drug Interaction
GEO: Gene Expression Omnibus
GM: Gut Microbiome
LB-CADD: Ligand-Based Computer-Aided Drug Design
MASI: Microbiota- Active Substance Interaction Database
MDAD: Microbe Drug Association Database
ODC: Ornithine Decarboxylase
OMIM: Online Mendelian Inheritance in Man
PAMPs: Pathogen-associated Molecular Patterns
PRR: Pattern Recognition Receptor
QSAR: Quantitative Structure-activity Relationships
RV: Reverse Vaccinology
SB-CADD: Structure-Based Computer-Aided Drug Design
TCEs: T Cell Epitopes
